# Effect of Plasma Treatment on Titanium Surface on the Tissue Surrounding Implant Material

**DOI:** 10.3390/ijms22136931

**Published:** 2021-06-28

**Authors:** Hitomi Tsujita, Hiroshi Nishizaki, Akiko Miyake, Seiji Takao, Satoshi Komasa

**Affiliations:** 1 Faculty of Health Sciences, Osaka Dental University, 1-4-4, Makino-honmachi, Hirakata-shi, Osaka 573-1144, Japan; tsujita@cc.osaka-dent.ac.jp (H.T.); nisizaki@cc.osaka-dent.ac.jp (H.N.); miyake-a@cc.osaka-dent.ac.jp (A.M.); 2 Department of Removable Prosthodontics and Occlusion, Osaka Dental University, 8-1, Kuzuhahanazono-cho, Hirakata-shi, Osaka 573-1121, Japan; takao-s@cc.osaka-dent.ac.jp

**Keywords:** osseointegration, dental implant, titanium, atmospheric pressure plasma treatment, hydrophilicity, tissue differentiation

## Abstract

Early osseointegration is important to achieve initial stability after implant placement. We have previously reported that atmospheric-pressure plasma treatment confers superhydrophilicity to titanium. Herein, we examined the effects of titanium implant material, which was conferred superhydrophilicity by atmospheric-pressure plasma treatment, on the surrounding tissue in rat femur. Control and experimental groups included untreated screws and those irradiated with atmospheric-pressure plasma using piezobrush, respectively. The femurs of 8-week-old male Sprague-Dawley rats were used for in vivo experiments. Various data prepared from the Micro-CT analysis showed results showing that more new bone was formed in the test group than in the control group. Similar results were shown in histological analysis. Thus, titanium screw, treated with atmospheric-pressure plasma, could induce high hard tissue differentiation even at the in vivo level. This method may be useful to achieve initial stability after implant placement.

## 1. Introduction

Many researchers have shown that surface modification of implant materials influences bone formation and maintenance at the interface, and thus plays a major role in osseointegration [1,2,3,4,5]. Numerous reports have investigated the correlation between the material surfaces and various cells [6,7,8,9,10,11]. Changes in the surface roughness of the material surface have been reported to contribute to increased bone marrow cell adhesion and differentiation. It has also been reported that when there is an increase in the surface roughness of the titanium surface, which is one of the main materials for implant materials, the initial adhesion, proliferation, differentiation of bone-related cells, and protein adsorption also increases [12,13,14,15]. Komasa et al. reported that titanium and nanostructured zirconia/alumina composite (NANOZR) implant materials modified by ultraviolet (UV), atmospheric-pressure plasma, and alkali treatments can promote the early formation of hard tissue in the tissue surrounding the implant [16,17,18,19,20,21,22,23,24,25]. Other commonly used surface-treatment methods include acid etching, sandblasting, anodization, physical vapor deposition, calcium phosphate coating, and hydroxyapatite coating [1,2,3,4,5,6,7,8,9,10,11].

Hydrophilization is known as a surface treatment method for titanium materials that can be performed by clinicians [26,27,28]. Superhydrophilic treatment of the material surface enhances the adhesion of cell adhesion proteins and bone proteins [29,30]. It has been reported that osseointegration, promotion of suppression of epithelial adhesion and bacterial adhesion of soft tissue around the implant are induced, and clinical applications are highly expected. Examples of physical modification methods for imparting hydrophilicity to the surface of a material include low-temperature atmospheric pressure plasma method and ultraviolet (UV) irradiation method. Chemical modification methods include the hydrogen peroxide solution immersion method and NaOH solution treatment method [31,32]. However, the methods that have attracted the attention of clinicians are the physical modification methods, such as low-temperature atmospheric-pressure plasma treatment and UV treatment, which have a low risk of material contamination. In the low-temperature atmospheric-pressure plasma method, free molecules are dissociated by a weak electric field under low voltage to generate plasma. For example, under atmospheric pressure, plasma processing produces ionized electrons and cations, as a high voltage is applied to the air [33,34,35,36]. Ozone is generated from plasma, and the redox reaction by active oxygen generated from plasma causes decomposition of organic matter and produces hydroxyl groups. In the UV irradiation method, oxygen radicals generated by photocatalysis are immediately oxidized at the molecular binding sites of organic compounds, cleaved by UV irradiation, to generate hydroxyl groups. These reactions activate the surface and improve the adhesiveness and wettability of the surface by Van der Waals force. Although few studies have examined the usefulness of both the methods, according to the latest research using the Quartz Crystal Microbalance (QCM) system, Matsumoto et al. examined it as a treatment for titanium surfaces [37]. When observed over time immediately after dropping onto the surface of the material, it was clarified that the material involved in osseointegration showed high adhesive strength on the surface of the plasma-treated titanium surface [37].

The range of applications for plasma treatment continues to expand, and it has been applied not only in the dental field but also in various other fields. Various papers have examined how plasma treatment of titanium surface affects the tissue surrounding the implant [38,39,40]. The effect of plasma treatment is to make hydrophobic materials hydrophilic by the effects of ablation and surface activity on the material surface. Although the effect of plasma treatment on the material surface has been clarified, the large size of the device is disadvantageous to clinicians. The device used in this study is piezobrush. Since this device is a handy type, it is useful in daily clinical practice. In past reports, Ujino et al., have shown that treating titanium with this device removes contaminants on the surface of the material and imparts hydrophilicity [41]. It was also clarified that this surface induces initial adhesion and differentiation induction of rat bone marrow cells (RBMCs) [41]. In addition, Takao et al., and Zeng et al., have already used this device and clarified the effect of plasma treatment on both titanium and NANOZR, which is expected to be used for patients with metal allergies [42,43]. However, for clinicians to use piezobrush with confidence, the effects of atmospheric pressure plasma treatment on the tissues surrounding the implant must be examined at the in vivo level. Further studies are needed on how atmospheric pressure plasma treatment exerts an effect on implant materials at in vitro and in vivo levels.

This study examined the effect of titanium screws plasma treated with piezobrush on the tissue surrounding the implant.

## 2. Results

### 2.1. Sample Preparation

**Figure 1:**
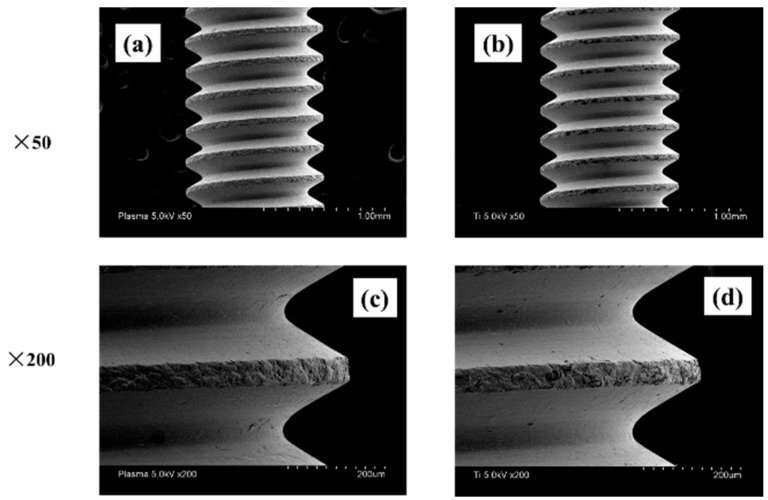
SEM images of titanium screw are shown. (plasma-treated samples; (**a**,**c**), untreated samples; (**b**,**d**) When the implant surface was observed at low (×50) and high (×200) magnifications, no mechanical change was observed on the material surface due to the plasma treatment.

**Figure 2:**
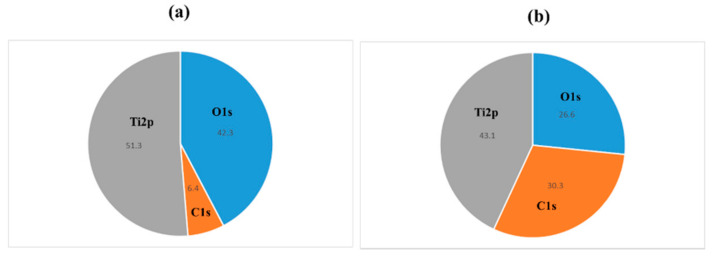
XPS analyses of the titanium screw are shown. Plasma-treated samples; (**a**), untreated samples; (**b**). By applying atmospheric pressure plasma treatment, an increase in oxygen peak and a decrease in carbon peak on the implant surface were observed.

**Figure 3:**
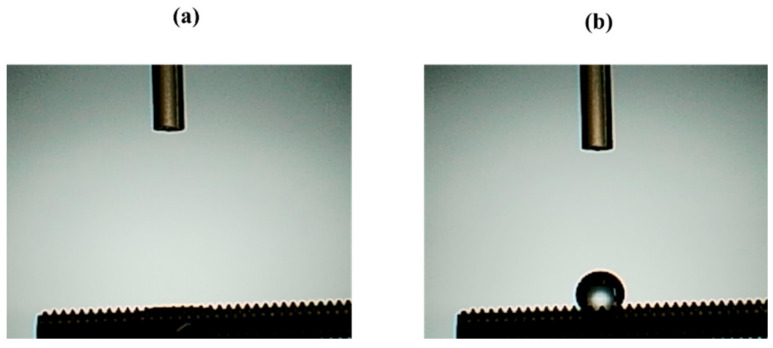
Image of the surface of the implant surface to which distilled water is dropped is shown. Plasma-treated samples; (**a**), untreated samples; (**b**). When the contact angle was measured, the untreated implant surface showed hydrophobicity, whereas the plasma-treated implant surface showed superhydrophilicity.

It was investigated how the atmospheric pressure plasma treatment on the titanium surface would affect the surface of the material. The Scanning electron microscopy (SEM) analysis results are shown in Figure 1. No difference was observed in the surface structure of the titanium screw due to plasma treatment. The X-ray photoelectron spectroscopy (XPS) analysis results are shown in Figure 2. The test group showed a reduction in carbon, which indicates a pollutant on the surface of the material. Figure 3 shows the results of the contact angle on the surface of the titanium screw. The contact angle of the test group showed 32°, while the experimental group showed superhydrophilicity.

### 2.2. Cell Morphology

**Figure 4:**
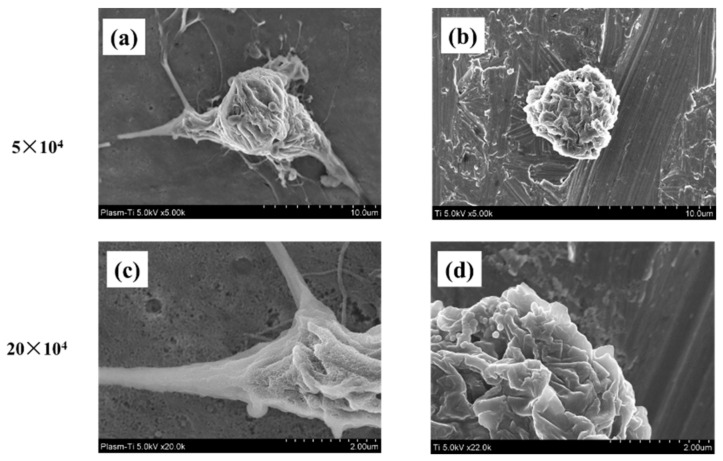
SEM images of RBMCs on titanium disks are shown. Plasma-treated samples; (**a**,**c**) untreated samples; (**b**,**d**). Adhesion of RBMCs was observed on titanium surface with or without plasma treatment. No extension of the cell process of RBMCs was observed on the untreated titanium surface. However, changes in the morphology of RBMCs were observed by plasma treatment. When observed at high magnification, prolongation of the cell process was observed.

The morphology of the RBMCs attached to the surface of the titanium disk was observed 6 h after culturing (Figure 4). Changes were observed in the shape of the cells seeded on titanium surface depending on the presence or absence of atmospheric pressure plasma treatment. The oval shape of RBMCs was clearly observed on the untreated titanium disk. No filamentous pseudopodia were observed during the observation of strong enlargement. The morphology of RBMCs had the shape of a spindle in the form of a titanium surface treated with atmospheric-pressure plasma. Observations of strong enlargement revealed that filamentous pseudopodia were acquired. It was clarified that cell adhesion was promoted by applying atmospheric pressure plasma treatment.

### 2.3. Intracellular ROS Level for RBMCs

**Figure 5:**
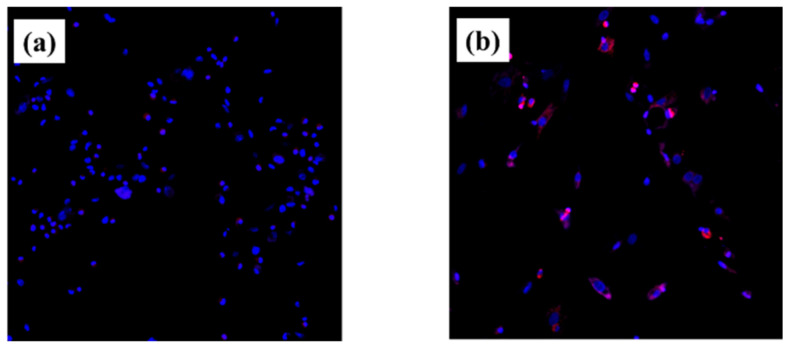
ROS images of RBMCs on titanium disks are shown. Plasma-treated samples; (**a**) untreated samples; (**b**). Adhesion of RBMCs was observed in the form of titanium surface in both groups. (Blue; DNA of RBMCs) ROS was observed in RBMCs on the untreated titanium samples. (Pink; ROS) On the other hand, the presence of ROS was not recognized in titanium surface treated with atmospheric pressure plasma.

No intracellular Reactive Oxygen Species (ROS) in RBCs were observed on the plasma-treated titanium surface. That is, it was shown that by performing the plasma treatment, active oxygen is generated on the surface of the material, and an environment in which cells can easily grow is formed (Figure 5).

### 2.4. Plasma-Induced Bone Differentiation on Titanium Surfaces In Vivo

**Figure 6:**
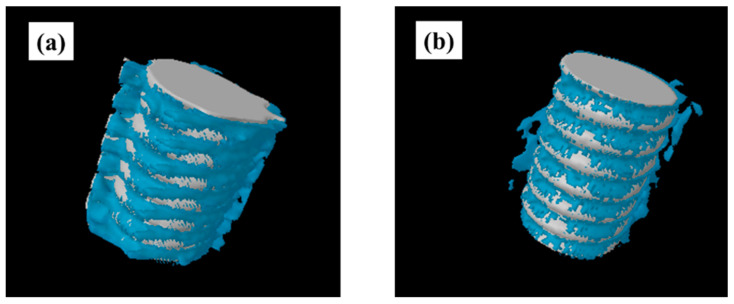
CT images of titanium screw around the rat femur removed 8 weeks after implantation are shown. Plasma-treated samples; (**a**) untreated samples; (**b**) It was revealed that the plasma-treated implants showed CT images with a large amount of new bone formation.

**Figure 7:**
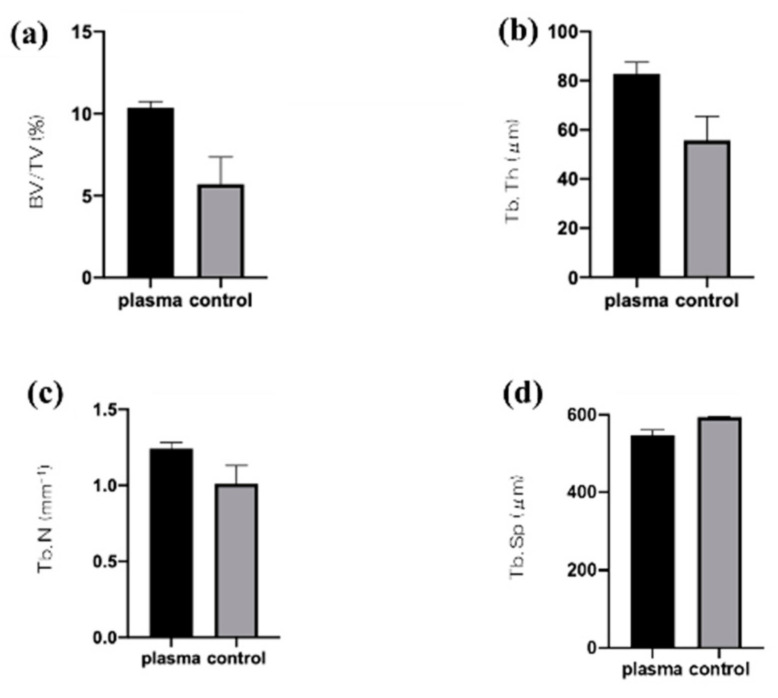
Bone volume (BV)/tissue volume (TV) (**a**), mean trabecular number (Tb.N) (**b**), and mean trabecular thickness (Tb.Th) (**c**) were significantly higher in the test implant group compared to the control implant group (*p* < 0.05). Conversely, mean trabecular separation (Tb.Sp) (**d**) exhibited a significantly lower value in the plasma-treated titanium implants compared with the untreated implants.

Figure 6 shows the results of analysis of the rat femur removed 8 weeks after implantation by three-dimensional computed tomography (CT). It was revealed that the test group showed CT images with a large amount of new bone formation. Quantitative evaluation within the region of interest (ROI) is shown in Figure 7. From ROS analysis results, the amount of new bone around the implant was significantly higher than that in the untreated group with titanium screw treated with atmospheric pressure plasma. (Figure 7).

**Figure 8:**
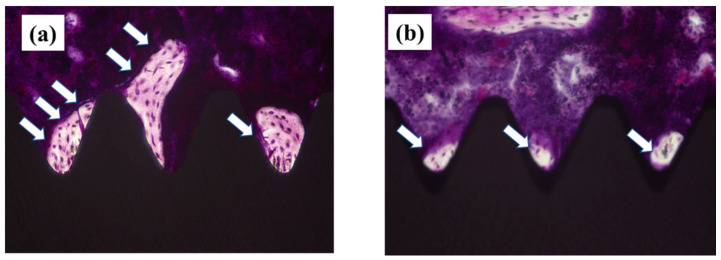
Histopathological image of bone tissue around the implant 8 weeks after implantation. Plasma-treated samples; (**a**) untreated samples; (**b**) The part indicated by the white arrow indicates the new bone. As shown in the figure, it is shown that the amount of new bone formed is large around the plasma-treated implant screw.

**Figure 9:**
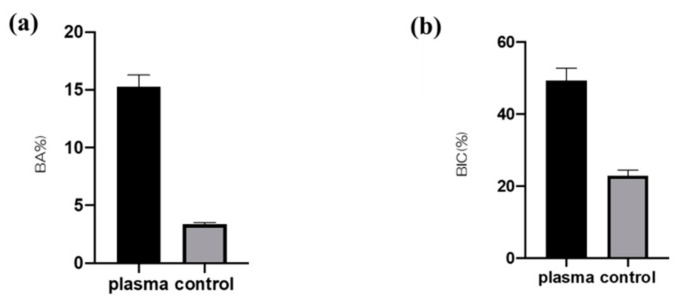
Analysis of newly formed bone around the implants in the plasma-treated and untreated titanium screws. Using quantitative histomorphometric analysis of bone area ratio (BA) (**a**) and bone-to-implant contact (BIC) (**b**) from the results of both measurements, it was clarified that the amount of new bone formed was large on the surface of the plasma-treated implant material.

A non-decalcified specimen prepared from the femur in which the implant is placed is shown in Figure 8. As shown in Figure 8, it was clarified that the amount of new bone formed was large on the surface of the plasma-treated titanium implant screw. The result of the quantitative analysis performed in this measurement area is shown in Figure 9. The bone area (BA) and bone implant contact (BIC) values calculated by quantitative analysis were significantly higher in the experimental group (*p* < 0.05).

**Figure 10:**
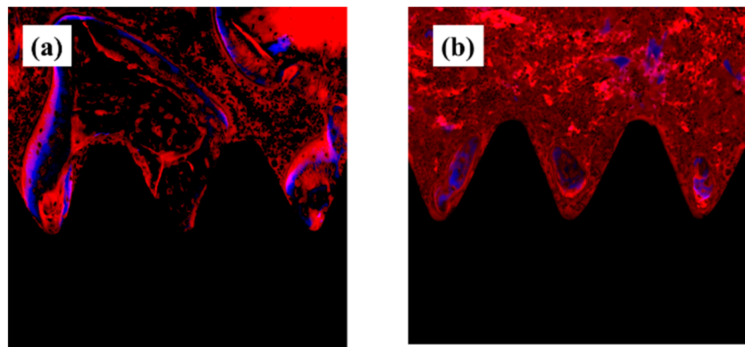
Fluorescent label and dynamic tissue morphometry was observed with a confocal laser scanning microscope. Plasma-treated samples; (**a**) untreated samples; (**b**). The red linear signal indicates 4 weeks after implantation and the blue linear signal indicates 8 weeks after implantation. The new bone mass, indicated by the distance between the lines, was shown to be high on the plasma-treated titanium implant surface.

**Figure 11:**
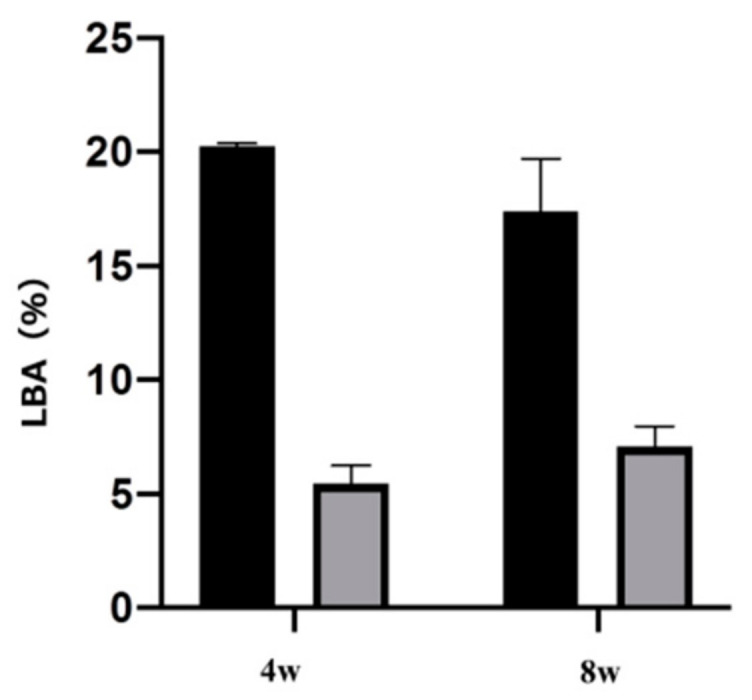
The analysis result of the bone area stained with the labeled antibody is shown in the figure. Both analysis results at 4 and 8 weeks after implantation revealed that the atmospheric pressure plasma treatment group had a large amount of new bone formation (*p* < 0.05).

Fluorescent labels were injected 4 and 8 weeks after implantation, and dynamic tissue morphometry was observed with a confocal laser scanning microscope. Colored linear signals representing alizarin red S (4 weeks) and oxytetracycline hydrochloride (8 weeks) were shown. Observation of the distance between the fluorescently stained new bones at each measurement time showed that the amount of new bone formed on the plasma-treated titanium implant screw surface was high in all of the weeks (Figure 10). In addition, quantitative analysis of this analysis image showed that the proportion of bone area (percentage of labeled bone area; %LBA) stained with the labeled antibody was high in the experimental group. In addition, it is suggested that the plasma-treated group has a large amount of new bone formed relatively early (Figure 11).

## 3. Discussion

In previous studies, we have shown that at the in vitro level, plasma treatment using piezobrush on the surface of titanium metal contributes to the formation of hard tissue in the tissue surrounding the implant. When this method was applied to a titanium implant screw and implanted in the rat femur, it was revealed that a significant amount of new bone was formed around the surface of the implant material. When the titanium surface treated with atmospheric pressure plasma was examined, hydrophilicity was imparted to the material surface, oxidative stress on the titanium surface was reduced, and elongation of cell processes was observed. Adding previous reports, this result suggests that atmospheric pressure plasma treatment is a method that can be recommended to clinicians.

Characteristics of the implant surface has profound influence not only on cell adhesion and development, but also on cell differentiation and expression. Generally, a rough surface has a stronger cell adhesion force than a mirror surface. In our previous reports, we showed that the concentrated alkali treatment of titanium forms a nanometer-scale mechanical structure on the titanium surface, which is helpful for advancing the initial adhesion ability of RBMCs and the ability to induce hard tissue differentiation [16,17,18]. At the same time, it is said that the surface texture of the material surface is greatly involved in the initial adhesion and kinetics of RBMCs. Among the surface properties, hydrophilicity is said to have a large correlation with cell adhesion. Many studies have reported that the initial adhesion, proliferation, and differentiation of cells tend to increase on the surface of highly hydrophilic materials [1,2,3,4,5,6,7,8,9,10]. Generally, UV treatment and atmospheric pressure plasma treatment are examples of clinical methods that can impart hydrophilicity to the surface of the implant material [44,45,46,47]. Among these, atmospheric pressure plasma treatment is often used for material modification by utilizing the phenomenon of ionizing electrons and cations, mainly by applying a high voltage to oxygen. Accelerated electrons cleave the interatomic bonds of the oxygen molecules to generate active oxygen. The mechanism of atmospheric pressure plasma treatment is that this active oxygen decomposes hydrophobic organic substances adsorbed on the surface of the material and gives the surface of the material superhydrophilicity. In our previous reports, it was reported that applying atmospheric pressure plasma treatment using piezobrush to the titanium surface is useful at the in vitro level for improving the initial adhesion of RBMCs and the ability to induce hard tissue differentiation [41,42,43,44]. In this study, the effect of atmospheric pressure plasma treatment on titanium surfaces was investigated for evaluation in vivo. Similar to the report by Ujino [41], the surface structure of the titanium screw did not change, and C was observed to indicate the state of contamination on the surface, indicating that the material surface was superhydrophilic. Plasma treatment introduces hydrophilic functional groups onto the surface of the main titanium and zirconia material as implant materials. Various cells and proteins involved in osseointegration adhere to the surface of the implanted implant material. It has been reported that these substances are easily adsorbed on the surface of the material to which hydrophilicity is imparted. Conditions for imparting hydrophilicity to the surface of the material include removal of contaminants on the surface and impartation of surface energy. Compared to UV treatment, atmospheric pressure plasma treatment has stronger plasma energy, so it is said that the amount of organic pollutants removed is large. Similar results were shown in the results of this experiment, and it is presumed that the plasma treatment removed contaminants from the surface of the implant screw and imparted hydrophilicity. A titanium surfaces must always maintain a clean surface to accelerate osseointegration. ROS has been reported in various reports as being capable of decomposing organic pollutants adsorbed on the surface of materials. Because implant treatment involves surgical procedures, the inflammation that occurs in the tissue surrounding the implant is said to be due to oxidative stress. [48,49]. Excessive amounts are known to cause cell apoptosis and delayed tissue healing, resulting in implant treatment failure. The presence of oxidative stress can prevent the formation of new bone in the tissue surrounding the implant placement. Conversely, suppressing oxidative stress provides a good growing environment for RBMCs. In this study, it was clarified that oxidative stress was reduced because active oxygen was formed on the titanium surface by plasma treatment. Thus, it is inferred that the change in the initial behavior of RBMCs was caused by ROS suppression, removal of pollutants, and impartation of superhydrophilicity. In this experiment, the initial behavior of RBMCs was investigated using SEM observation of a titanium surface seeded with RBMCs [40,50,51]. On the untreated material surface, RBMCs showed a spherical morphology. However, elongation of cell protrusions was observed on the surface of the material treated with atmospheric-pressure plasma. It has been reported that information transmission to the cell nucleus accompanying structural changes in the actin cytoskeleton is important in the process of differentiation from RBMCs to osteoblasts. The results of this study are similar, and together with the previous reports, it can be considered that the titanium surface subjected to atmospheric pressure plasma treatment is suitable as a growth environment for RBMCs. In this study, the titanium surface is treated with atmospheric pressure plasma to remove stains on the surface of the material and reduce oxidative stress to create an environment in which cells can grow easily. It was clarified from SEM analysis using RBMCs that this is effective in promoting adhesion of RBMCs.

The rat femur model used in the in vivo evaluation of this study is for evaluating the formation of bone tissue at the interface where the implant surface contacts the cancellous bone. The period of new bone formation in rats is about 8 weeks, and the period of this study is appropriate. Since the initial reaction of the implant surface contacting the bone tissue is thought to be involved in the subsequent osseointegration, this time we evaluated it for 4 weeks. Therefore, this study was conducted in two stages, 4 to 8 weeks after implantation [21,40,42,43]. It is clear from various studies that even at the in vivo level, the bone bond on the surface of the implant material that was imparted with hydrophilicity, was better than that on the surface of the untreated implant material. This study indicated high neoplastic bone formation with titanium screws treated using atmospheric-pressure plasma, in all in vivo evaluations. Ujino et al., revealed that titanium plate treated with atmospheric pressure plasma improved the expression level of markers related to the induction of hard tissue differentiation [27]. New bone formation on the surface of plasma-treated implant material is closely related to the initial bone reaction and is considered to be consistent with that of in vivo analysis. A definite difference was observed in the new bone formation at 4 weeks. As shown in Ujino et al. [27], there is a clear difference in the genetic markers of calcification 3 to 4 weeks after the instillation of RBMCs on the surface of the material. It is speculated that this increasing tendency of the calcification rate induces a difference at the in vivo level. As mentioned above, in vitro, and in vivo studies are closely correlated, and changes in the initial behavior of bone marrow cells on the surface of the material in our previous reports are those of new bone in the tissue surrounding the implant. It is considered to be involved in an increase in the amount of formation. No infection was observed in any of the rats used in this experiment, and it was clarified that they have antibacterial properties while maintaining high ability to induce hard tissue differentiation. From a long-term vision, this method can be expected to be useful as a modification device for implant materials. However, for this material to be truly useful in clinical settings, studies using large animals such as beagles are required, and ultimately its application to humans must be considered. We believe that piezobrush will be an important device for clinicians because it is small and relatively simple, and will definitely help in dental clinics. 

In previous studies, we have shown at the in vitro level that plasma treatment using piezobrush on the surface of pure titanium metal contributes to the formation of hard tissue in the tissue surrounding the implant.

## 4. Materials and Methods

### 4.1. Sample Preparation

The experimental group was divided into two groups: the atmospheric-pressure plasma-treated group and the untreated group. Titanium samples (JIS Grade 2, 15 mm in diameter and 1 mm-thick, Daido Steel, Osaka, Japan) of in vitro and titanium screw implants (1.2 mm in external diameter and 12 mm in length, Daido Steel, Osaka, Japan) were used in this study. Plasma treatments on titanium surface were performed using a piezobrush^®^ PZ2 (Relyon Plazma GmbH, Regensburg, Germany). Plasma treatment was performed using active gas in atmospheric-pressure, low-temperature plasma treatment under irradiation (0.2 MPa) for 30 s at 10 mm. Scanning electron microscope (SEM, S-4800; Hitachi, Tokyo, Japan) and a scanning probe microscope (SPM, SPM-9600; SHIMADZU, Kyoto, Japan) are used for observing the titanium surface. X-ray photoelectron spectroscopy (XPS) (Kratos Analytical Axis Ultra DLD electron spectrometer; Kratos Instruments, Manchester, UK) are used for analyzing the component of the samples. Contact angle measurements of the test and control implants were performed using a video contact-angle measurement system (SImage Entry 6; Excimer Inc., Kanagawa, Japan). The measurement was performed after 2.6 μL of distilled water were dropped immediately after the surface treatment of titanium implants.

### 4.2. Cell Culture

Animal experiments in this study were performed in accordance with the guidelines for animal experimentation at Osaka Dental University (approval no. 20-08001). RBMCs were obtained from the femurs of 8-week-old Sprague-Dawley (SD) rats (SHIMIZU Laboratory Supplies Co., Kyoto, Japan). The method for establishing a primary culture of bone marrow cells from rat femur followed our previous report. Third-generation bone marrow cells were seeded in plasma-treated and untreated titanium. The method for establishing a primary culture of bone marrow cells from rat femur followed our previous report.

### 4.3. Cell Morphology

RBMCs were seeded on specimens at a density of 4 × 10^4^ cells/cm^2^. Samples were washed, in which a cell was stuck by phosphate-buffered physiological saline (PBS), fixed by 4% glutaric aldehyde and dehydrated by a step-by-step ethanol series after 6 h of culture. The form of the cell stuck on plasma treated and untreated titanium surface was observed by SEM.

### 4.4. Cell Intracellular ROS Level of RBMCs

Intracellular ROS levels were determined using the CellROX^®^ oxidative stress reagent (C10422, Thermo Fisher Life Technologies Ltd., Tokyo, Japan). ROS levels on RBMCs of the test and control titanium disks were stained and observed by confocal laser scanning microscopy (LSM700, Carl Zeiss, City, Zeiss, Germany).

### 4.5. Rat Distal Femur Model In Vivo

In this study, 20 male SD rats (Shimizu Laboratory Supplies Co., Kyoto, Japan; age 8 weeks, weighing 160 ± 15 g) were used (the test group; 10 rats, the control group; 10 rats). In vivo analysis in this study was based on our previous study [21,24,42,43]. Animals were administered inhalation anesthesia, followed by intraperitoneal injection of anesthetics (1.5 mL/kg). The hair was shaved off at the right hind limb, and the skin was disinfected with iodine, followed by cleaning with 75% ethanol to remove the iodine. A 1 cm long longitudinal skin incision was made along the medial side of the knee joint, and the subcutaneous fascia was incised. The patella and extensor mechanisms were then dissected to expose the distal aspect of the femur. A pilot hole was drilled through the intercondylar notch using a 1 mm-round dental burr under profuse sterile saline irrigation, and the hole was enlarged to 1.2 mm with an endodontic file. The implants, sterilized by ethylene oxide gas, were randomly inserted into the 20 prepared channels and the medullary cavities of the right femur. After surgery, the knee joint was restored, and the surgical site was closed in layers. The animals received intramuscular injections of gentamicin (1 mg/kg) and buprenorphine (0.05 mg/kg) for 3 days to prevent postsurgical infection and relieve pain. All rats were allowed free movement without any restrictions.

### 4.6. Sequential Fluorescence Labeling

Polychrome sequential labeling of bone using intraperitoneal injection of fluorochromes was performed to record the process of new bone formation and mineralization after the implantation, according to the following schedule: week 4, alizarin red S at 30 mg/kg (Sigma-Aldrich, St. Louis, MO, USA), and week 8, oxytetracycline hydrochloride at 25 mg/kg (Sigma-Aldrich, St. Louis, MO, USA). All animals were sacrificed by an intraperitoneal overdose of sodium pentobarbital 3 days after the final labeling treatment.

### 4.7. Plasma-Induced Bone Differentiation on the TNS-Modified Titanium Surface In Vivo

Immediately after dissection, the right femurs, including the implants, were placed in a cool saline solution, and scanned with a micro-computed tomography scanner (microCT, SkyScan 1275, Bruker, Kontich, Belgium), operated at 100 kV and a pixel size of 10 μm in all spatial directions. After tomographic acquisitions, the implant and surrounding tissue were reconstructed and analyzed using morphometric software (TRI/3D-BON; Ratoc System Engineering, Tokyo, Japan). The ROI was defined as the 500 μm-wide area of bone around the implants from 2 mm below the highest point of the growth plate to the distal 100 slices [32]. The bone volume fraction (BV/TV), mean trabecular number (Tb.N), mean trabecular thickness (Tb.Th), and mean trabecular separation (Tb.Sp) were calculated within the ROI.

After the micro-CT scan, the femoral specimens were used to create undecalcified histological sections. The specimens were fixed in 70% ethanol solution for 7 days, followed by immersion in Villanueva bone stain solution. The sections were histomorphometrically analyzed using a BZ-9000 digital microscope (Keyence Co., Osaka, Japan). Fluorescence microscopy was also performed using a confocal laser scanning microscope (LSM 700, Carl Zeiss, Jena, Germany). The excitation/emission wavelengths of the chelating fluorochromes were 351/460 nm, 543/617 nm, and 488/517 nm for alizarin red S (red) and oxytetracycline hydrochloride (blue), respectively. The region of measurement was defined on sections approximately 2 mm below the growth plate to 1 mm distally, according to the micro-CT analysis.

### 4.8. Statistical Analysis

**Figure 12:**
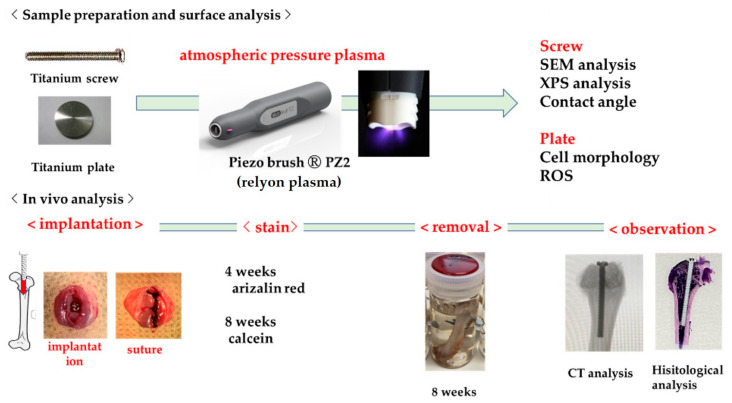
The study design is shown. Experiments can be divided into two areas. One is to confirm how the atmospheric pressure plasma treatment on titanium surface affects the material surface. The other is an in vivo analysis using rat femur. Four weeks after implantation, alizarin red was injected as a labeled antibody, calcein was injected at eight weeks, and the patient was euthanized. The femur was removed together with the implant and CT analysis and histological analysis were performed.

Four replicates of all samples were prepared. Data are presented as the mean ± standard deviation. In all analyses, statistical significance was determined using the paired two-tailed Student’s *t*-test. Statistical significance was set at *p* < 0.05. The study design was designed as shown in Figure 12.

## 5. Conclusions

In addition to the previous papers, the results of this experiment revealed that the amount of new bone formation in the tissue surrounding the implant was increased by applying atmospheric pressure plasma treatment to titanium screw. The reason for this is that atmospheric pressure plasma treatment improves the wettability of the material surface and reduces ROS. As a result, it was clarified that the improvement of the adhesive strength of RBMCs attached to the material surface was induced, which seems to be related to the result shown in the in vivo evaluation. Since this device is lightweight and easy to use, it can be expected to be a device that can be recommended to clinicians.

## Data Availability

The data presented in this study are available on request from the corresponding author.
